# Preparation of a Facilitated Transport Membrane Composed of Carboxymethyl Chitosan and Polyethylenimine for CO_2_/N_2_ Separation

**DOI:** 10.3390/ijms14023621

**Published:** 2013-02-07

**Authors:** Jiang-Nan Shen, Chang-Chao Yu, Gan-Ning Zeng, Bart van der Bruggen

**Affiliations:** 1 College of Chemical Engineering and Materials Science, Zhejiang University of Technology, Hangzhou 310014, China; E-Mails: ycc198818@hotmail.com (C.-C.Y.); gnzeng@zjut.edu.cn (G.-N.Z.); 2 Laboratory for Applied Physical Chemistry and Environmental Technology, Department of Chemical Engineering, K.U. Leuven, W. de Croylaan 46, Leuven B-3001, Belgium; E-Mail: bart.vanderbruggen@cit.kuleuven.be

**Keywords:** facilitated transport, blend membrane, carboxymethyl chitosan, polyethylenimine, carbon dioxide

## Abstract

The miscibility of carboxymethyl chitosan/polyethylenimine (CMCS/PEI) blends was analyzed by FT-IR, TGA and SEM. Defect-free CMCS/PEI blend membranes were prepared with polysulfone (PSf) ultrafiltration membranes as support layer for the separation of CO_2_/N_2_ mixtures. The results demonstrate that the CMCS/PEI blend is miscible, due to the hydrogen bonding interaction between the two targeted polymers. For the blended membrane without water, the permeability of CO_2_ gas is 3.6 × 10^−7^ cm^3^ cm^−2^ s^−1^ cmHg^−1^ and the corresponding separation factor for CO_2_ and N_2_ gas is about 33 at the pressure of 15.2 cmHg. Meanwhile, the blended membrane with water has the better permselectivity. The blended membrane containing water with PEI content of 30 wt% has the permeance of 6.3 × 10^−4^ cm^3^ cm^−2^ s^−1^ cmHg^−1^ for CO_2_ gas and a separation factor of 325 for CO_2_/N_2_ mixtures at the same feed pressure. This indicates that the CO_2_ separation performance of the CMCS/PEI blend membrane is higher than that of other facilitated transport membranes reported for CO_2_/N_2_ mixture separation.

## 1. Introduction

Carbon dioxide (CO_2_) is an acidic gas, which must be removed from raw natural gas to prevent corrosion of the pipeline. CO_2_ recovery from flue gas (primarily in mixtures with N_2_) is becoming more and more important due to regulations controlling emissions of greenhouse gases. Membranes aiming at selectively removing CO_2_ from gas mixtures have therefore attracted great interest. As a result, it is becoming an emerging technology in natural gas upgrade, landfill gas recovery, enhanced oil recovery and global warming prevention [1]. Compared to traditional acid gas treatment technologies, membrane technology offers inherent advantages, such as its small footprint, mechanical simplicity, and high energy efficiency. The membranes prepared by current commercial polymer materials suffer from the tradeoff between permeability and selectivity suggested by Robeson [2]. In the development of polymer materials, the facilitated transport membranes (FTMs) offer potential to elevate the upper boundary between permeability and selectivity for gas separation through the reversible reactions between reactive carriers of membrane materials and the target gas, such as carbon dioxide in this case [3–6].

There are two main types of reactive carriers: the mobile carrier, which can move freely across the membrane, and the fixed carrier, which only has very limited mobility around its equilibrium position. In mobile carriers containing membranes, the mobile carriers react with a specific component on the feed side forming the carrier-solute complexes; these complexes move across the membrane and release the specific component on the down-stream side. Although this type of membrane shows a remarkably high selectivity [7], membrane instability caused by carrier solution evaporation especially at a high temperature or carrier solution being forced to permeate through the porous support under a high transmembrane pressure, and carrier degradation led by the irreversible reaction with impurities in the feed gas stream [8]. In fixed carriers containing membranes, the specific component reacts at one carrier site, when carriers are bonded fundamental polymer chains by covalent bond, washout and evaporation of carrier are effectively prevented [9,10].

Since it is a tedious part of the design of a new material for membrane synthesis, polymer blending offers several advantages such as simplicity, reproducibility and commercial viability, resulting in a new polymer with synergetic properties [8,9]. Compared to other modification technologies or new preparation materials, blending of polymers is preferred for membrane synthesis [11]. In our previous work, a CMCS/poly (vinyl alcohol) (PVA) blend membrane was prepared for pervaporation [12]. The prepared membrane displayed a good separation performance for ethanol/water mixtures because the membrane possesses many hydroxyl and carboxyl groups. Matsuyama *et al.* [9] reported a CO_2_ facilitated transport membrane consisting of PEI as a fixed carrier and PVA as a plasticizer. The results show that only CO_2_ was transported by the facilitated transport mechanism and that PEI functioned efficiently as the carrier of CO_2_. Hamouda *et al.* [13] prepared a (PVA)/PEI/poly(ethylene glycol) (PEG) membrane and investigated its CO_2_/N_2_ separation performance. A higher membrane selectivity towards CO_2_ via PEG sorption and phase separation of PEG in the PVA/PEI/PEG matrix was not acquired. However, these blended membranes were stable due to both their high hydrophilicity and the retention of PEI by entanglement with PVA chains. Xing [14] reported a PVA/PEG membrane for CO_2_ capture. The results show that PEG is miscible with PVA, as shown by SEM imaging. Thus, the studies mentioned above indicate that the PEG/PEI blend is immiscible. In attempting to find an explanation, PEG/PEI blends were prepared in our laboratory; macro phase separation was clearly observed due to the fact that solvation molecular dynamics partially screening out the repulsive interaction between solvated chain segments are removed upon drying, so that repulsive polymer segmemt-segment interaction may prevail [13]. The repulsive interaction may be caused by the electon-rich [15].

Polyethylenimine contains primary and second amino groups that can react with CO_2_ reversibly. The ratio of primary amino groups, secondary amino groups and tertiary amino groups in PEI is approximately equal to 1:2:1. Since it is difficult to form membranes with the pristine PEI, blending with other polymers was envisaged. It was expected that CMCS would be appropriate because it possesses a large number of reactive carboxymethyl (–CH_2_COO^−^) and amino (–NH_2_) groups. These functional groups can interact with the groups of amino groups with PEI by formation of hydrogen bonds.

Chitosan, a derivative of chitin, is similar in chemical structure to cellulose and is the second abundant biopolymer after cellulose to be found in nature. However, it is not developed and applied to the extent of cellulose materials. Chitosan membranes have good thermal properties and they have a proven performance for the separation of CO_2_/N_2_ and CO_2_/O_2_ [16–21]. However, the disadvantage of chitosan is that it is insoluble in water at neutral pH, which restricts its application. Based on these findings, CMCS is proposed to be used for CO_2_ capture. Zhang [22] prepared pure CMCS membranes for separation of CO_2_/CH_4_ mixtures. The CO_2_ permeation flux is 5.44 × 10^−5^ cm^3^ cm^−2^ s^−1^ cmHg^−1^ and the separation factor is 33.8, which is better than that of typical chitosan membranes.

In this paper, we followed Hamouda and Matsuyama’s idea of enhancing CO_2_ separation properties by introduction of PEI into PVA. It is expected that entanglement of to obtain the entanglement of the polymeric carrier with CMCS chains through crosslinking. Acetic acid treated CMCS/PEI blend membranes were prepared, and their gas separation performance for CO_2_/N_2_ mixtures was investigated.

## 2. Experimental

### 2.1. Chemicals

**Scheme 1:**
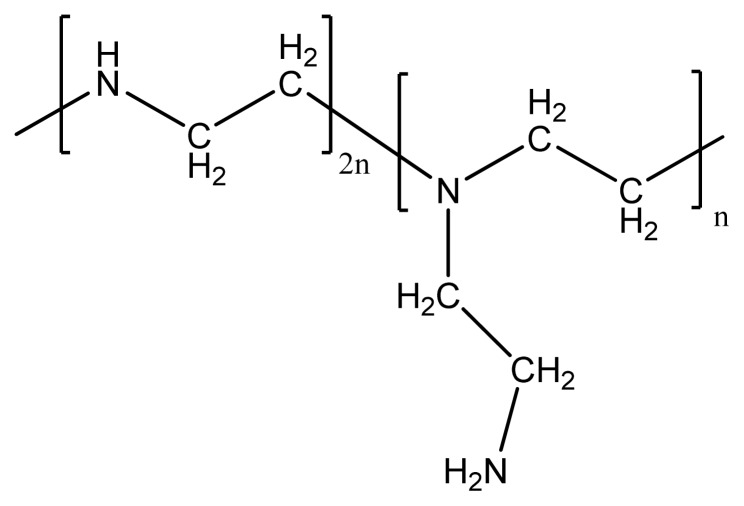
Molecular structure of PEI.

**Scheme 2:**
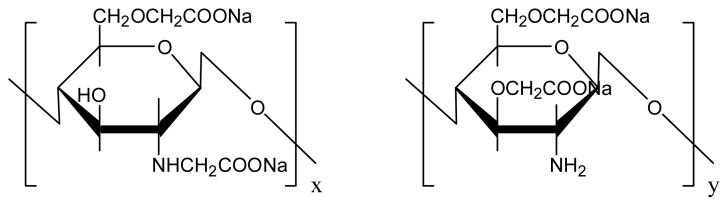
Molecular structure of CMCS.

PEI (MW 60,000) was purchased from Acros Organics. Scheme 1 shows the molecular structure of PEI. CMCS with a substitution degree of 90% and an average molecular weight of 6 × 10^5^ was purchased from Zhejiang Aoxing Biotechnology Co. Ltd., China. The molecular structure of CMCS is shown in Scheme 2. Deionized water was generated by electrodialysis in the laboratory with tap water as input. The PSf UF membrane (MWCO = 30,000) was supplied by National Engineering Research Center for Liquid Separation Membranes, China. All other reagents and chemicals were of analytical grade.

### 2.2. Membrane Preparation

Membranes for separation tests were prepared by solution casting and the solvent evaporation technique. Five grams of CMCS was dissolved in 95 mL of deionized water. PEI aqueous solutions of 5 wt% were prepared by dissolving the PEI in deionized water. The composition of the CMCS/PEI blend solution was determined by adjusting the weight ratio of the above mentioned solutions. The composition of membrane casting solutions is shown in Table 1.

Then the solution was filtered to remove any undissolved and suspended matter. The cast solution used for casting the membranes was vacuumed to remove the bubble that might be trapped. The membrane was prepared by casting the polymer solution on a polysulfone ultrafiltration membrane as the support layer. Before casting, the PSf support membrane was rinsed with dilute NaOH solution (1 wt% of the NaOH concentration), and flushed with deionized water to remove any remained NaOH. Afterwards, membrane casting and its evaporation was followed at room temperature for at least 24 h. And then, the dry membrane was kept in the thermostatic chamber at 40 °C, which was filled with saturated acetic acid vapor to crosslink for 10 to 50 min [23]. The desired thickness of the membrane was adjusted by a casting knife. The resulted membrane thickness is about 16 μm as measured by scanning electron microscope (SEM).

### 2.3. Membrane Characterization

The miscibility of binary polymer blends composed of CMCS and PEI was investigated by using TGA (TGA-7, PerkinElmer, Norwalk, CT, USA), Fourier transform infrared reflection (FT-IR) (Nicolet6700, Madison, WI, USA) and scanning electron microscope (SEM) (Hitachi S4700A, Tokyo, Japan). The FT-IR spectrums of blend samples were recorded in the region of 4000–1000 cm^−1^. The scanning resolution is 4 cm^−1^. The sample was treated by the KBr-pellet technique. SEM images of membranes were taken after being dried in a vacuum oven, and then coated with gold. The thermal behavior of membrane materials (CMCS/PEI = 40/60, PEI, CMCS) was analyzed by using TGA. The temperature range used was from 30 °C to 700 °C, and the heating rate was 10 °C/min.

### 2.4. Permeation Experiments

**Figure 1:**
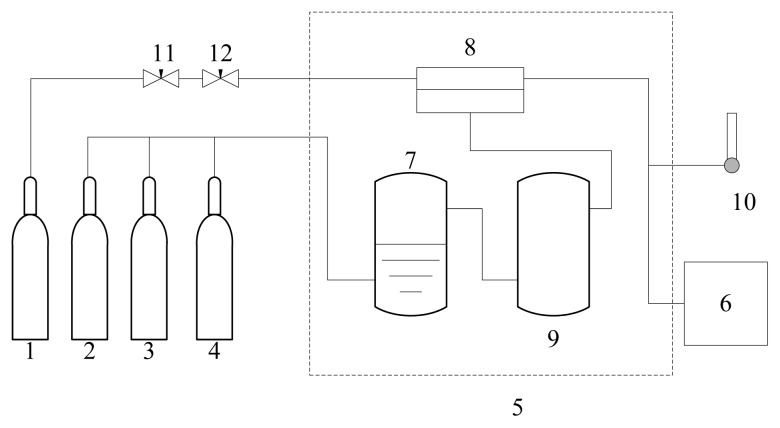
Experimental setup for gas permeation measurements (1. H_2_ 2. CO_2_/N_2_ mixture 3. CO_2_ 4. N_2_ 5. Constant temperature bath 6. GC 7. Humidifier 8. Test cell 9. Condense water remover 10. Flow meter 11. Gas pressure maintaining valve 12. Gas flow maintaining valve).

The permeation testing set-up used is shown in Figure 1. The effective area of the tested composite membrane is 19.6 cm^2^. The gas permeance of the membrane without water was tested with dry feed gas (these are denoted as dry membranes). For the membrane containing water, the feed gas was humidified by a humidifier (these are denoted as humidified membranes). The resulting membranes were tested with a feed gas that was either a pure gas, or a mixture of CO_2_ and N_2_ (CO_2_/N_2_ = 1/9). The permeance of the gas was calculated from the flow rate of H_2_ (used as the sweep gas) and the peak area of CO_2_ and N_2_, measured in a gas chromatograph (Shimadzu GC1024, Kyoto, Japan) with a thermal conductivity detector. The downstream pressure in the apparatus was 1 atm. The permeance was calculated as *R*_i_ = *N*_i_/Δ*p*_i_, and the ideal separation factor (for pure gas) and sepration factor (for mixed gas) are determined by *R*_CO2_/*R*_N2_.

In these definitions, *N*_i_ is the permeation flux of permeate gas, Δ*p*_i_ is the trans-membrane partial pressure difference, and *R*_CO2_ and *R*_N2_ are the permeance of CO_2_ and N_2_, respectively.

The swelling degree (SD) of the cross-linked membrane was calculated by the following equation:

(1) $$SD(\%)=((W_{\text{s}}-W_{\text{d}})/W_{\text{d}})\times 100$$

where *W*_s_ and *W*_d_ refer to the weight of the dry and the swollen cross-linked blends.

## 3. Results and Discussion

### 3.1. Miscibility of the CMCS/PEI Blends

**Figure 2:**
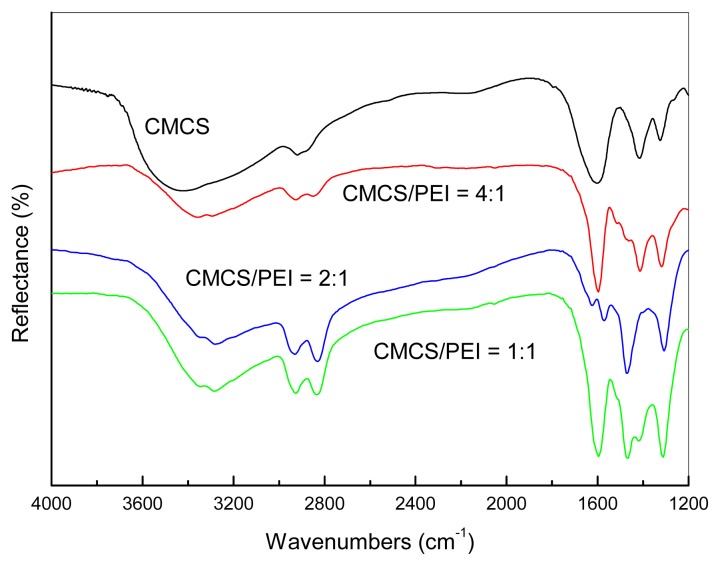
IR spectrums of the membrane materials.

The FT-IR spectrums of the CMCS and the CMCS/PEI blends (CMCS(*w*)/PEI(*w*) = 4/1, 2/1, 1/1) are shown in Figure 2. As shown in Figure 2, PEI nitrogen atoms are strong hydrogen bond acceptors (or Lewis bases), which must generate stronger hydrogen-bond interactions and blend miscibility with CMCS containing hydroxyl group. The broad O–H stretching peak of CMCS in the range from 3500 cm^−1^ to 3000 cm^−1^ splits into two components involving PEI. The two broad peaks of 3280 cm^−1^ and 3340 cm^−1^ represent the O–H stretching, stretching of H-bonded O–H and N–H stretching of primary and secondary amines of CMCS and PEI. Moreover, the broad band is distinct from the increase in PEI content. The better resolution of the broad band of strong H-bonds of CMCS hydroxyl in the range from 3000 to 3600 cm^−1^ into distinct peaks with the increase in PEI content in the CMCS/PEI blends is consistent with the hypothesis of strong H-bonds between CMCS and PEI. The results are similar to the previous reports [10], which indicate the existence of hydrogen bonds between CMCS and PEI leading to the miscibility of CMCS/PEI.

**Figure 3:**
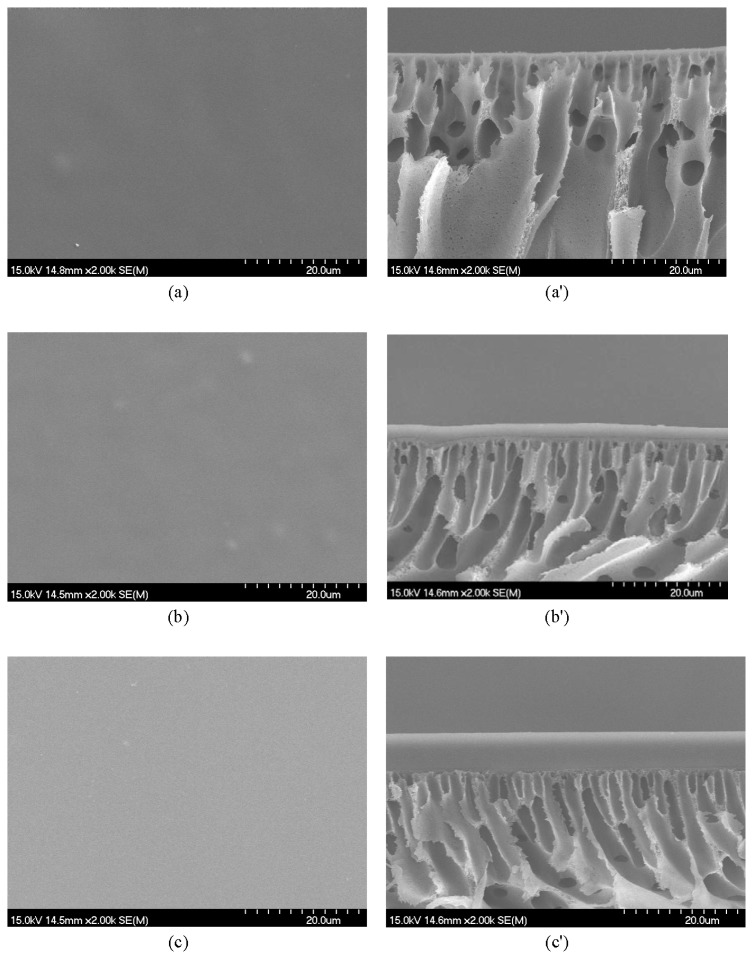
SEM views of the surface and cross section of the blend composite membranes (CMCS(w)/PEI(w) = a. 80/20, b. 60/40, c. 40/60, a, b, c surface and a′, b′, c′ cross section)

Figure 3 shows the SEM images of the surface and cross section of the blended membranes CMCS/PEI = 80/20, 60/40 and 40/60. It can clearly be seen that the blends have a smooth surface, similar to the blends of carboxylmethyl chitosan/alginate [23], also indicating the excellent miscibility between CMCS and PEI. From the scanning electron microscopy image of the cross section of different blended membranes, it can be seen that the separation layer of the composite membrane is dense and smooth, and all of the upper layer of the composite membranes, the functional blends, have a symmetric structure and the absence of nodules indicating phase separation.

**Figure 4:**
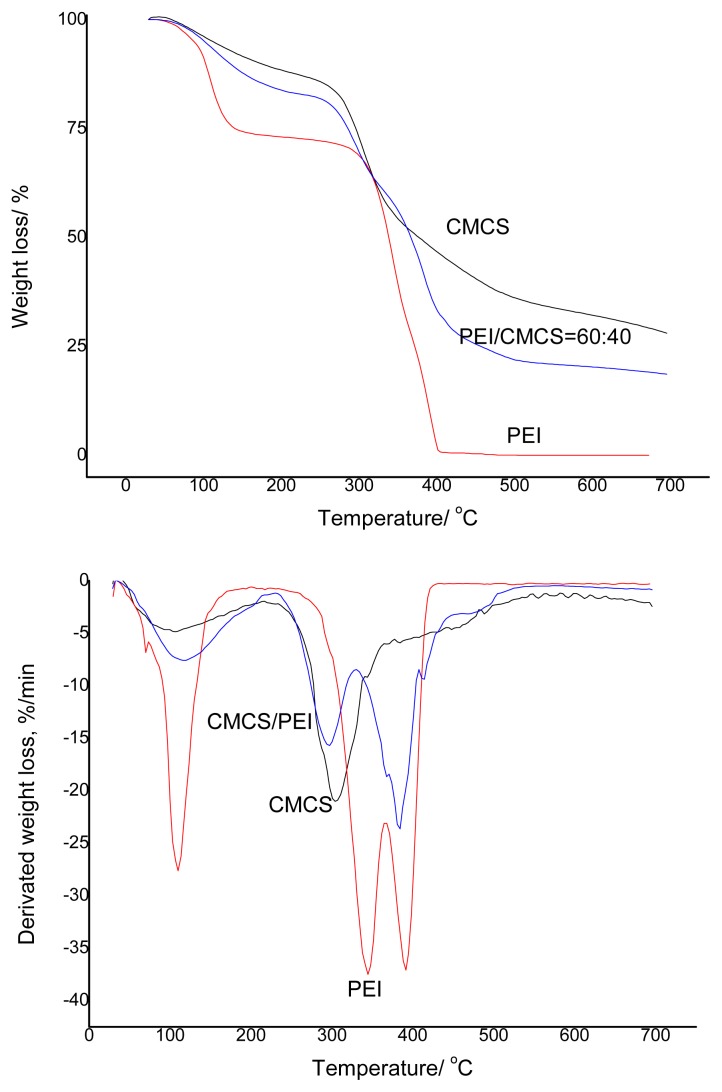
TGA-DTG thermograms of CMCS, PEI and CMCS/PEI.

The thermal stability of the blended membranes was performed by TGA. From Figure 4, the thermal degradation of the membrane can be observed. The blended membrane was proved to be stable at ca. 110 °C. However, a gradual loss of mass of the membranes was observed till 200 °C, followed by a sharp loss of mass of the membrane up to 345 °C. As for the pure CMCS membrane, 72% of the mass was lost at the decomposition temperature of 700 °C. Further, about 82% of the mass of the blended membrane is lost at the same temperature due to the low decomposition temperature of PEI polymer, which can be totally decomposes at 400 °C. Thus, if PEI is blended with CMCS, the eventual decomposition temperature is increased up to 700 °C, which is in line with other blended membranes [24]. This type of behavior may be due to the stronger interaction between the two polymers in the blend [25]. It was concluded from the FTIR, SEM and thermal analysis results that CMCS/PEI is miscible.

### 3.2. Effect of Feed Gas Pressure on the Performance of Non-Crosslinked Blended Membrane without Water

**Figure 5:**
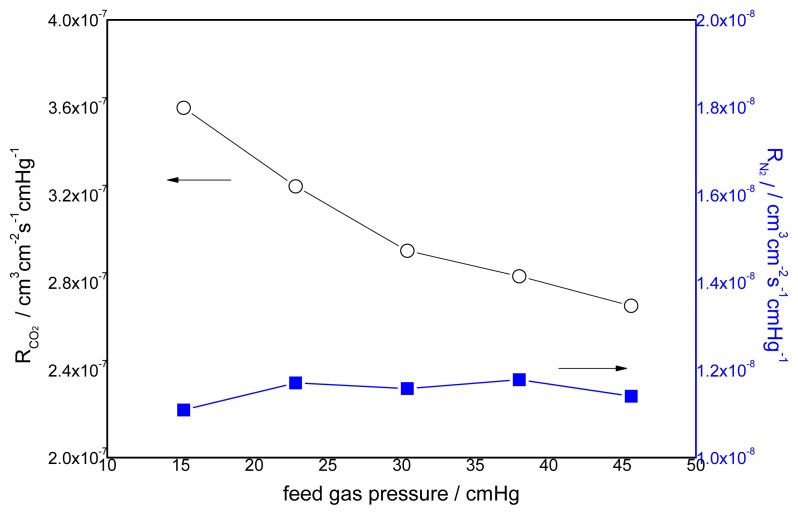
Effect of feed gas pressure on the gas permeation rate (pure gas) CMCS/PEI: 95/5, uncross-linked, 30 °C.

**Figure 6:**
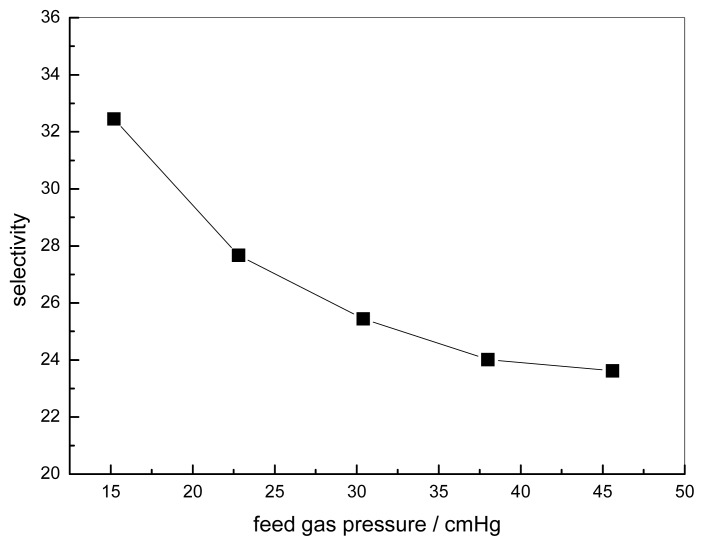
Effect of feed gas pressure on the ideal separation factor (pure gas) CMCS/PEI: 95/5, uncross-linked, 30 °C.

The effect of the feed gas pressure on the performance of the membrane is shown in Figure 5. The CO_2_ permeance decreases with increasing feed gas pressure, whereas the change of permeance of N_2_ is unclear. The different transport mechanism of CO_2_ and N_2_ in the obtained membranes causes a difference in permeation. Facilitated transport in the membrane is available to CO_2_ with the help of carriers. As one of the characteristics of facilitated transport, the membrane permeance will generally decrease at the beginning because of the increasing feed partial pressure due to the saturation of carriers [8]. N_2_ permeation follows the solution-diffusion mechanism, so the N_2_ flux varies linearly with the partial pressure of N_2_. This is because N_2_ has no chemical reactions with the carriers. Its sorption in the membrane can be described by Henry’s law, and the permeability is usually dependent on the feed pressure [26]. Therefore, the flux increases linearly with the feed pressure [27]. The effect of feed gas pressure on the membrane selectivity for CO_2_/N_2_ is shown in Figure 6. The selectivity decreases with increasing feed gas pressure. When the feed pressure is above 40 cmHg, the decreasing trend disappears and the selectivity of CO_2_/N_2_ becomes almost constant. For the blended membranes without water, the high permeance of CO_2_ is 3.6 × 10^−7^ cm^3^ cm^−2^ s^−1^ cmHg^−1^ and the ideal separation factor for the CO_2_/N_2_ mixture is about 33 at the pressure of 15.2 cmHg.

### 3.3. Effect of the Feed Gas Pressure on the Performance of Acid Treated Blended Membranes Containing Water

**Figure 7:**
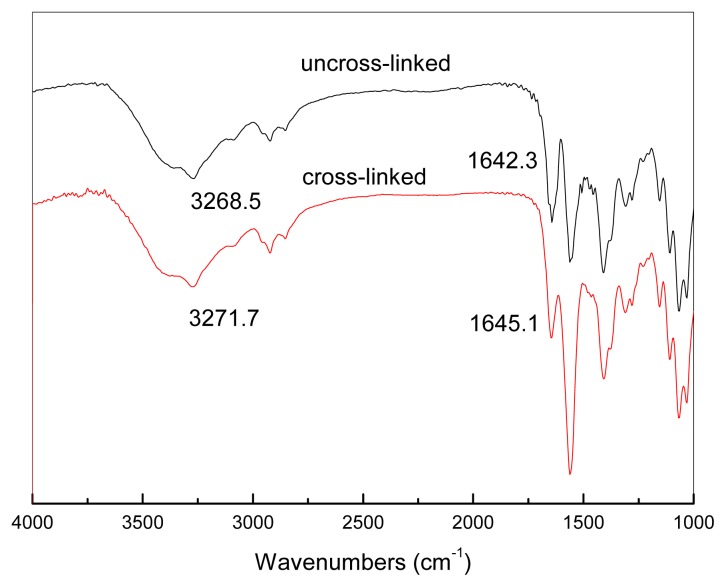
FTIR spectra of CMCS/PEI blend before and after treated by acetic acid.

**Figure 8:**
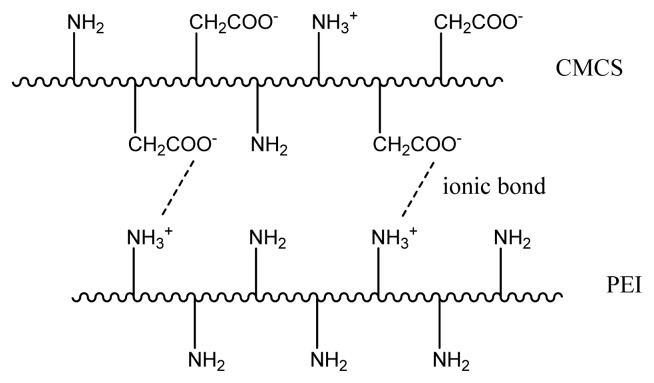
Protocol of cross-linked CMCS/PEI.

To enhance the permeance of the CMCS/PEI blend membrane, the feed gas was moist, which would cause defects in the water soluble blends. CMCS/PEI blends cross-linked by application of acid would be resistant to water and therefore more stable in a feed gas containing water. The bonds formed by cross-linking were studied by FTIR. Figure 7 shows the FTIR spectra of the uncross-linked and cross-linked CMCS/PEI blends. Unlike the cross-linking reactions initiated by cross-linking agents, the acid treatment reactions are relatively weak [28]. In the spectrum of acid treated CMCS/PEI, no evident new peaks were observed. The peak at 3268.5 cm^−1^ was attributed to N–H being shifted to a higher wavelength, which is 3272 cm^−1^, causing the ionization of free –NH_2_ in the process of acid treatment. There is no peak at around 1700 cm^−1^ assigned to C=O in carboxylic groups, which proves that there is no remaining acetic acid in the blend. The newly formed –NH_3_^+^ has an ionic bond with –COO^−^ leading to the peak at 1642 cm^−1^. The wavelength was attributed to C=O in ionized carboxylic group moving to higher wavelength of 1645 cm^−1^. To confirm the existence of the cross-linking points further, the acid treated CMCS/PEI blend was swollen in a solvent containing 50% (*v*/*v*) alcohol and 50% (*v*/*v*) water. As a result of swelling, the degree of swelling of CMCS/PEI blends before and after acid treatment are 57.8% and 13.1%, respectively. To investigate whether or not the ionic bonds were formed in the acid treated, a symmetric CMCS film was prepared and treated by acetic acid; it is found that the treated CMCS was soluble in the water and a homogenous solution was formed. It can be speculated that ionic bonds among segments of CMCS were weak or naught, which was not the vital origin of cross-linkage in acid treated CMCS/PEI blends. The protocol of cross-linked membrane is shown in Figure 8.

**Figure 9:**
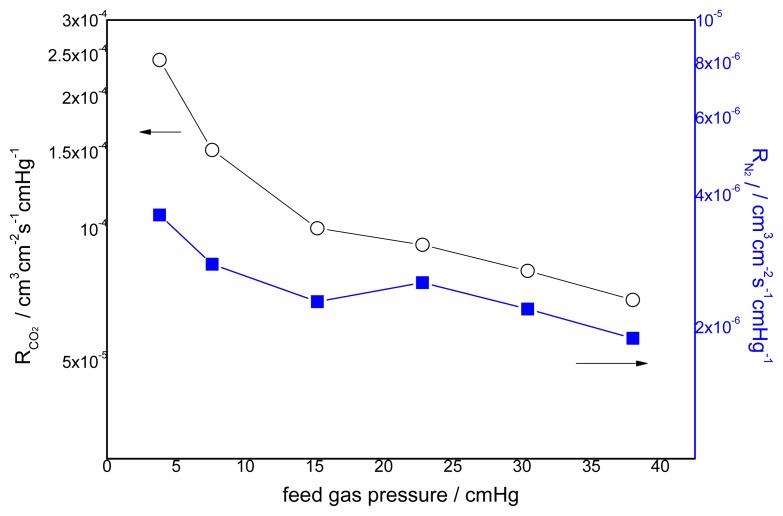
Effect of feed gas pressure on the gas permeation rate (CO_2_/N_2_ mixture) CMCS/PEI: 90/10 containing water, acid treatment for 10 min, 30 °C.

Due to the acid treatment, membranes were thought to be stable, and therefore the separation performance of the humidified feed gas was measured. The relative humidity was about 50%. The effect of the pressure of the feed gas containing water on the permeance of CMCS/PEI membrane treated by acetic acid is shown in Figure 9. It can be seen that the permeance of CO_2_ increased when using water as the catalyst in the reversible reaction of facilitated transport. In another case, the permeance of N_2_ was also enhanced, which can be caused by CO_2_ and water molecules acting as a plasticizer, increasing the free volume of the cross-linked blends.

In this case, the permeance of N_2_ was decreased as the increase of feed gas pressure. It was speculated that the membrane was denser as the feed pressure became higher. Zhang *et al.* [15] reported a supported liquid membrane using water as a carrier to separate CO_2_ from CO_2_/CH_4_ mixtures. They found that the water is important in facilitated transport of CO_2_ through membrane. In the wet membrane, CO_2_ is transformed into the small and easy-to-move ion HCO_3_^−^, and the CO_2_ transport is enhanced by carriers. Furthermore, the membrane is swollen by water resulting in enhancing the diffusion coefficient of CO_2_ in the membrane [29].

**Figure 10:**
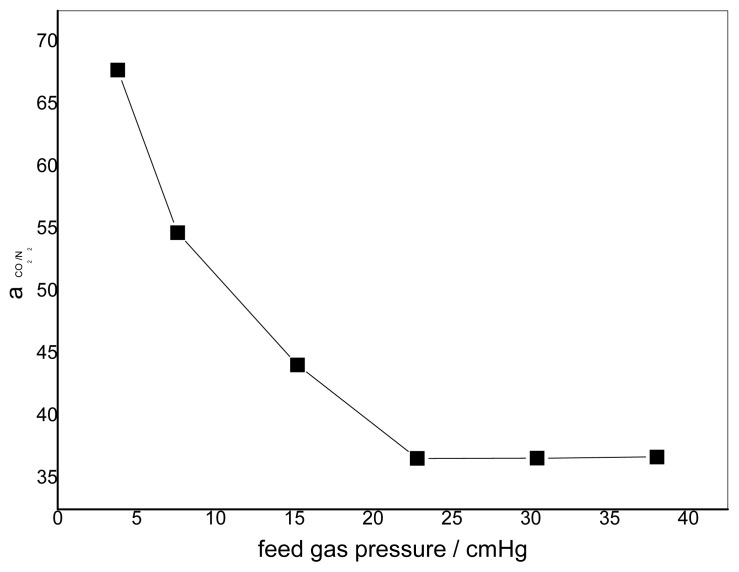
Effect of feed gas pressure on the separation factor (CO_2_/N_2_ mixture) CMCS/PEI: 90/10 containing water, acid treatment for 10 min, 30 °C.

Figure 10 shows the effect of the pressure of the feed gas containing water on the separation factor of CMCS/PEI for CO_2_ and N_2_. It was found that the separation factor decreased when the gas feed pressure increased, which is determined by the trend of CO_2_ permeance and N_2_ permeance. The separation factor of the humidified membranes is much larger than that of the un-humidified membranes.

### 3.4. Effect of the Duration of Acid Treatment on the Performance of Blended Membranes

**Figure 11:**
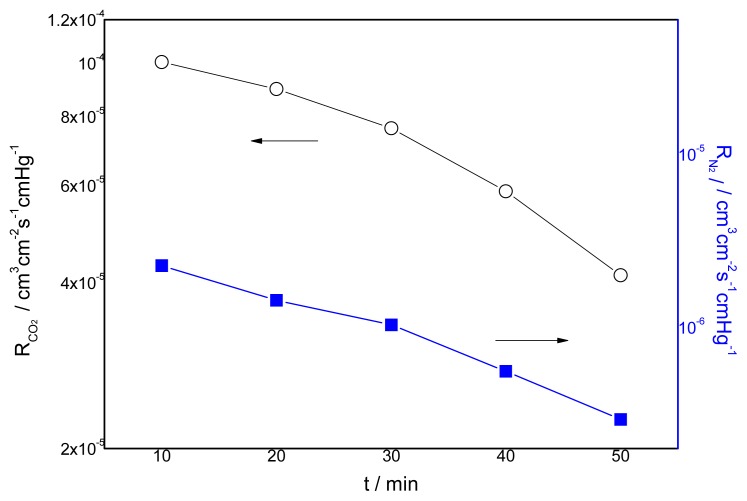
Effect of duration of acid treatment on gas permeation rate (CO_2_/N_2_ mixture) CMCS/PEI: 90/10 containing water, 15.2 cmHg, 30 °C.

**Figure 12:**
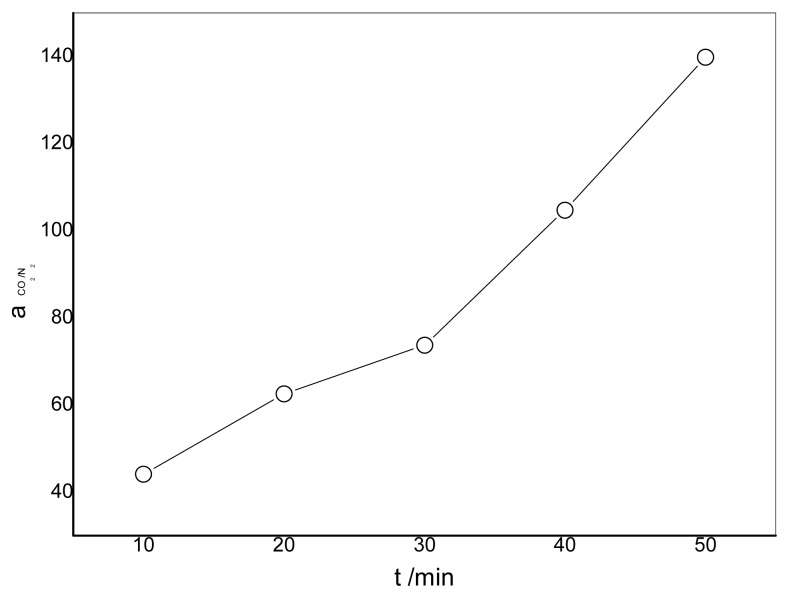
Effect of duration of acid treatment on separation factor (CO_2_/N_2_) CMCS/PEI: 90/10 containing water, 15.2 cmHg, 30 °C.

The effect of the duration of acid treatment on the membrane permeance is shown in Figure 11. It can be seen that the longer the duration of acid treatment, the lower the gas permeance will be. The longer the duration is, the more –NH_2_ will be ionized and the amount of cross-linking points between –NH_3_ and –COO^−^ will be larger. On the other hand, –NH_2_ is a carrier for the CO_2_ rather than –NH_3_. As a result, the decrease of –NH_2_ also causes a decrease in the permeance of CO_2_. The permeance of N_2_ gas decreases when the cross-linking time increases which results in the restriction of the motion of polymeric chains by the ionic bonds. Figure 12 presents the effect of the duration of acid treatment on the separation factor. It can be seen from Figure 12 that the separation factor increases when the duration is longer. The highest selectivity obtained was more than 140 for the membrane prepared with CMCS/PEI in a ratio of 90/10, which is much higher than that of the CMCS membrane.

### 3.5. Effect of the Content of PEI on the Performance of Blended Membranes

**Figure 13:**
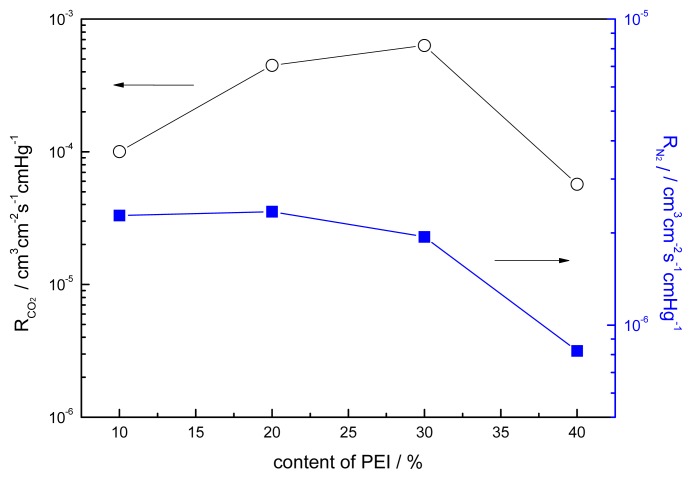
Effect of the content of PEI on the gas permeation rate (CO_2_/N_2_ mixture) CMCS/PEI: operation containing water, acid treatment for 10 min, 15.2 cmHg, 30 °C.

**Figure 14:**
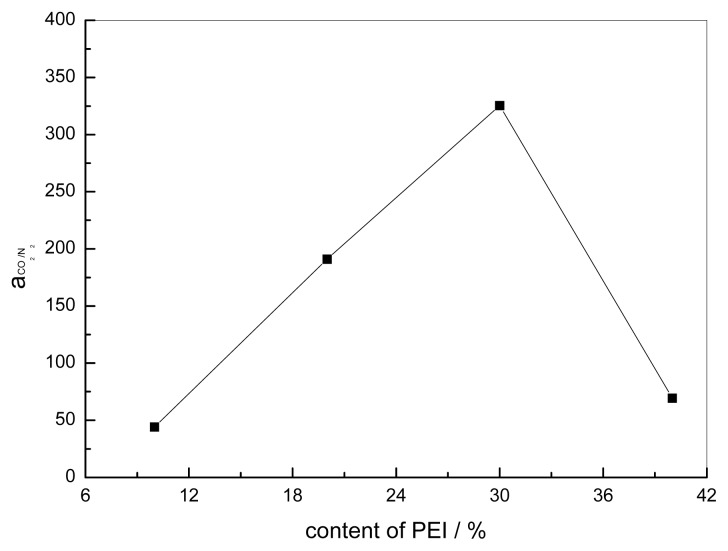
Effect of the content of PEI on the separation factor (CO_2_/N_2_ mixture) CMCS/PEI: operation containing water, acid treatment for 10 min, 15.2 cmHg, 30 °C.

The effect of the content of PEI on the gas separation permeance for CO_2_/N_2_ is shown in Figure 13 and Figure 14. The permeance of CO_2_ has a maximum value of 6.3 × 10^−4^ cm^3^ cm^−2^ s^−1^ cmHg^−1^ at a content of PEI of 30% and the separation factor is 325.4 at 15.2 cmHg. The explanation for this phenomenon is still not yet clear; for gas permeation the formation of crystals in polymer membranes is generally deleterious since they act as impermeable obstacles to gas molecular transport [28]. As for the fixed carrier membrane, crystallization will lead not only to a decrease of the effective permeation area but also to the decrease of the effective carrier content of the membrane. Matsuyama [9] reported that the maximum point was caused by the salting-out effect. Yi [30] prepared a PVAm/PEG membrane, and the crystallization of the blends of different contents of PEG was investigated. For gas permeation the formation of crystals in polymer membranes is generally deleterious since they act as impermeable obstacles to gas molecular transport [28]. As for the fixed carrier membrane, crystallization will lead not only to a decrease of the effective permeation area but also to the decrease of the effective carrier content of the membrane. The carriers in the crystallization region cannot make contact with the CO_2_ molecule. The decreasing permeance of N_2_ causes the crystallization of blends, which increases with the addition of PEI. The permeation and separation factor initially increased with the PEI content, reached a maximum, and then decreased with further increase of the PEI content. This can be explained by the larger contribution of the facilitated transport to the total permeation [13].

## 4. Comparison with Membrane Separation Performance Reported in the Literature

Table 2 shows the CO_2_ separation performance of membranes in this study and results of facilitated transport blend membranes reported in previous literature. These blended membranes have a favorable membrane performance due to the high hydrophilicity of the blend membrane and the retention of fixed carrier polymer by entanglement with hydrophilic polymer chains. Moreover, the separation performance of the CMCS/PEI blend membrane is comparable to or higher than other facilitated transport membranes reported for CO_2_/N_2_ separation.

The lack of stability has always been a major problem in facilitated transport membranes. The stability of the swollen membrane was carried out over a prolonged period of time. Throughout the experiments, the feed gas was humidified to retain the membrane permeability. There was no significant deterioration in the membrane properties in several weeks. This is because it has the highest hydrophilicity among the blended membranes and the immobilization of the PEI carrier in the blend structure by hydrogen bonds with CMCS, which probably resulted in the stability of the performance [10]. Our swollen blends membrane can be utilized for CO_2_ separations in flue gas purification and natural gas. There are many factors such as temperature, relative humidity, separation layer thickness and membrane preparation process that will influence the membranes performance. These parameters should be further studied to obtain the optimized conditions for the preparation of blended membranes with better performance.

## 5. Conclusions

FTIR, SEM and TGA observations indicate that CMCS and PEI are miscible. For the blended membranes without water, the permeance of CO_2_ is 3.6 × 10^−7^ cm^3^ cm^−2^ s^−1^ cmHg^−1^ and the ideal separation factor for pure CO_2_ and N_2_ gas is about 33 at a pressure of 15.2 cmHg. Acid treatment is necessary to crosslink the 90/10 CMCS/PEI blends; perfect membranes without defects were prepared. The permeance of CO_2_ through the acid treated 90/10 CMCS/PEI membrane containing water is 1.0 × 10^−4^ cm^3^ cm^−2^ s^−1^ cmHg^−1^ and the separation factor for the CO_2_/N_2_ mixture is 44.1 at a pressure of 15.2 cmHg. The CO_2_ permeance and separation factor are up to 6.3 × 10^−4^ cm^3^ cm^−2^ s^−1^ cmHg^−1^ and 325.4 at 15.2 cmHg when the content of PEI is 30% in the blends. Compared to the blended membranes with water, the separation process without water was not efficient.

## Figures and Tables

**Table 1:** Membrane casting solution composition.

Blend ratio of CMCS/PEI	90/10	80/20	70/30	60/40
PEI content (wt%)	10	20	30	40

**Table 2:** Comparison of membrane separation performance in current work and in other fixed site carrier membranes.

Membrane material	Selectivity/separation factor	Permeance (cm^3^ cm^−2^ s^−1^ cmHg^−1^) or permeability *	Δ*P* (cmHg)	Feed gas (CO_2_%) (*v*/*v*)	Ref.
PEI/PVA (32.7%/67.3%)	160	3.9 × 10^−6^	5.016	pure CO_2_ and N_2_	[9]
PEI/PEG/PVA (PEI: 45%)	100–250	P_CO2_:250 Barrer *	76	pure CO_2_ and N_2_	[13]
PVAm/PEG (90%/10%)	63.1	5.8 × 10^−6^	96	pure CO_2_ and CH_4_	[30]
PVAm	1000	5.1 × 10^−6^	152	Pure CO_2_ and CH_4_	[31]
PVAm/PVA (80%/20%)	174	2.1 × 10^−4^	152	CO_2_/N_2_ (10%)	[32]
PVAm/PVA contaiing water (80%/20%)	160	3.0 × 10^−4^	152	CO_2_/N_2_ (10%)	[33]
CS/PAm (50%/50%)	O_2_/CO_2_ 0.074	1.18 × 10^−11^	10	pure CO_2_ and O_2_	[16]
Swollen CS	70	2.5 × 10^−8^	25.4	CO_2_/N_2_	[18]
Swollen CS	CO_2_/N_2_ 250	4.8 × 10^−8^	15	CO_2_/N_2_/H_2_ (10/80/10)	[21]
Swollen CS/arginine salt (60%/40%)	CO_2_/N_2_ 852	1.5 × 10^−7^	11.4	CO_2_/N_2_/H_2_ (10/80/10)	[34]
Amino acid ionic liquid-based facilitated transport membranes	100	14,000 Barrer	76	pure CO_2_ and N_2_	[7]
Polyvinylalcohol (cross linked formaldehyde)	338	1728 Barrer	76~152	Gas Mixtures of 40/20/40 or 75/25/0 of H_2_, CO_2_ with N_2_	[10]
Dry CMCS/PEI (95%/5%)	33	3.6 × 10^−7^	15.2	pure CO_2_ and N_2_	This work
CMCS/PEI Containing water (70%/30%)	325	6.3 × 10^−4^	15.2	CO_2_/N_2_ (10/90)	This work

The units of permeance and pressure are converted to units used in the current paper (cm^3^ (STP)/cm^2^ s cmHg); ΔP represents the approximate partial pressure difference over the membrane (feed-permeate side). Selectivity is for pure gas, separation factor is for mixture;

* For Reference 10, the information of the membrane thickness is not obtained, so the permeability was used to show membrane performance.
